# Supplementary material for: PROTAC-Mediated GSPT1 Degradation Impairs the Expression of Fusion Genes in Acute Myeloid Leukemia
Source: Cancers (Basel). 2025 Jan 10;17(2):211. doi: 10.3390/cancers17020211 (PMC11763475; doi:10.3390/cancers17020211)
Supplement: Supplementary file 1 [file cancers-17-00211-s001.zip › Perzolli et al_ supplementary tables.pdf]

Name	Type	Supplier
DMSO	Dimethyl sulfoxide	Sigma Aldrich
Palbociclib	CDK4/CDK6 selective inhibitor	DC Chemicals
BSJ-03-123	CDK6 PROTAC	Activated Scientific
CST651	CDK6 PROTAC	*
GU3341	CDK6 PROTAC	*
CC-90009	GSPT1 PROTAC	MedChemExpress

TABLE S1: Drugs list used in this manuscript. * PROTACs provided by the Department of Pharmacceutical & Medicinal Chemestry at the University of Boon.

Genes	Forward/Reverse	Sequence
GAPDH	Fw	GAAGGTGAAGGTCGGAGTC
	Rev	GAAGATGGTGATGGGATTTC
TBP	Fw	CCTAAAGACCATTGCACTTCGT
	Rev	GTTCGTGGCTCTCTTATCCTCA
RUNX1::RUNX1T1	Fw	AATCACAGTGGATGGGCCC
	Rev	TGCGTCTTCACATCCACAGG
RUNX1	Fw	GCCTTCAGAAGAGGGTGCAT
	Rev	CTGGCATCGTGGACGTCTCTA
FUS::ERG	Fw	AGCAGTGGTGGCTATGAACC
	Rev	GGTGCCTTCCCAGGTGATG
ERG	Fw	AACCCTAGCCAGGTGAATGG
	Rev	ATAGCGTAGGATCTGCTGGC

TABLE S2: Forward and reverse primer sequences for RT-qPCR.

Antibody	Species raised	Dilution	Supplier	Catalog number
CDK6	Mouse	1:200	Santa Cruz Biotechnology	sc-7961
CDK4	Rabbit	1:1000	Cell Signaling	D963E
Phospho-Rb	Rabbit	1:1000	Cell Signaling	8180
IKZF1	Rabbit	1:500	Santa Cruz Biotechnology	sc-13039
GSPT1	Rabbit	1:1000	Sigma Aldrich	HPA052488
ERG 1/2/3	Rabbit	1:100	Santa Cruz Biotechnology	sc-271048

GAPDH	Mouse	1:5000	Hytest Ltd	5G4
Vinculin	Mouse	1:2000	Sigma	V9131
ß-tubulin	Rabbit	1:1000	Cell Signaling	2128S

TABLE S3: Antibodies list for immunoblotting
