# Supplementary material for: PROTAC-Mediated GSPT1 Degradation Impairs the Expression of Fusion Genes in Acute Myeloid Leukemia
Source: Cancers (Basel). 2025 Jan 10;17(2):211. doi: 10.3390/cancers17020211 (PMC11763475; doi:10.3390/cancers17020211)

A.

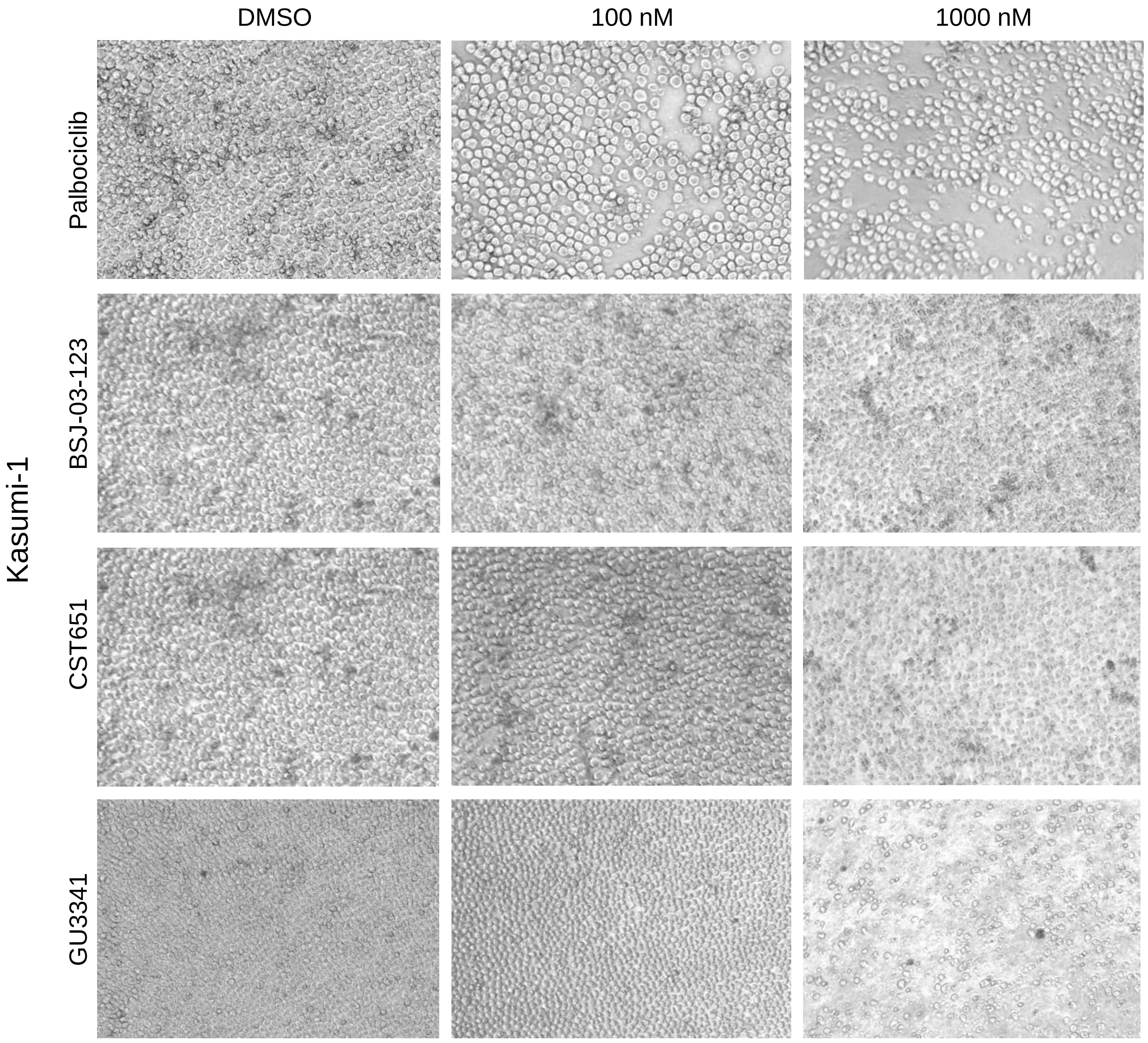

B.

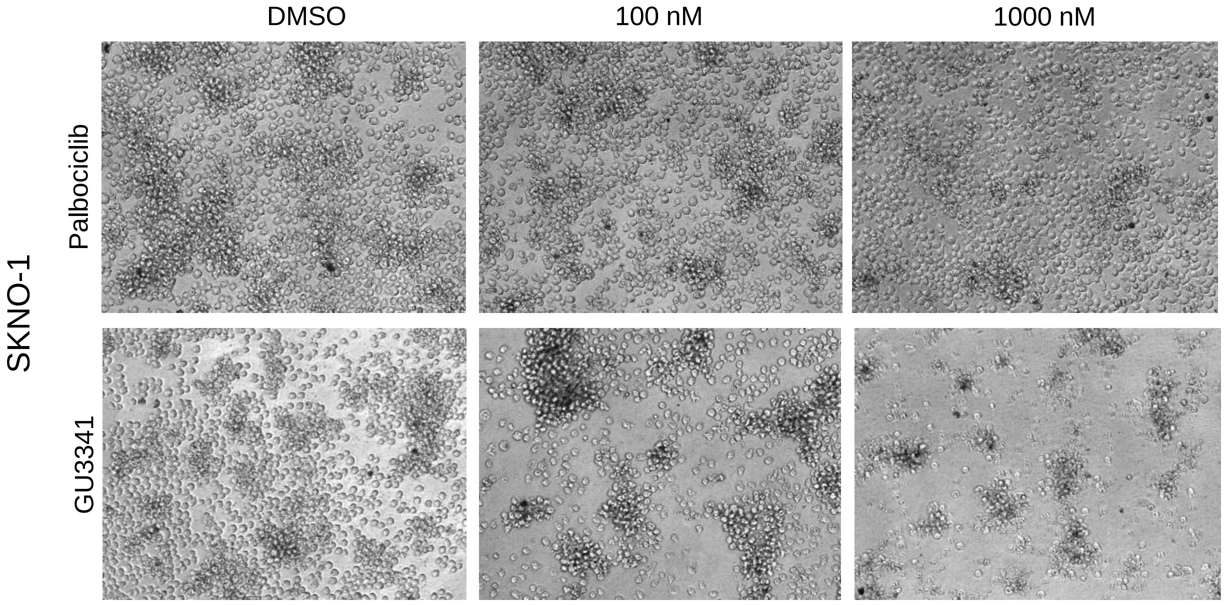

C.

**Kasumi-1**	**ED_{50} ± SD (nM)**
Palbociclib	15 ± 3
BSJ-03-123	42 ± 4
CST651	480 ± 3
GU3341	164 ± 2

D.

**SKNO-1**	**ED_{50} ± SD (nM)**
Palbociclib	11 ± 2
BSJ-03-123	11 ± 3
CST651	51 ± 3
GU3341	81 ± 2

Figure S1

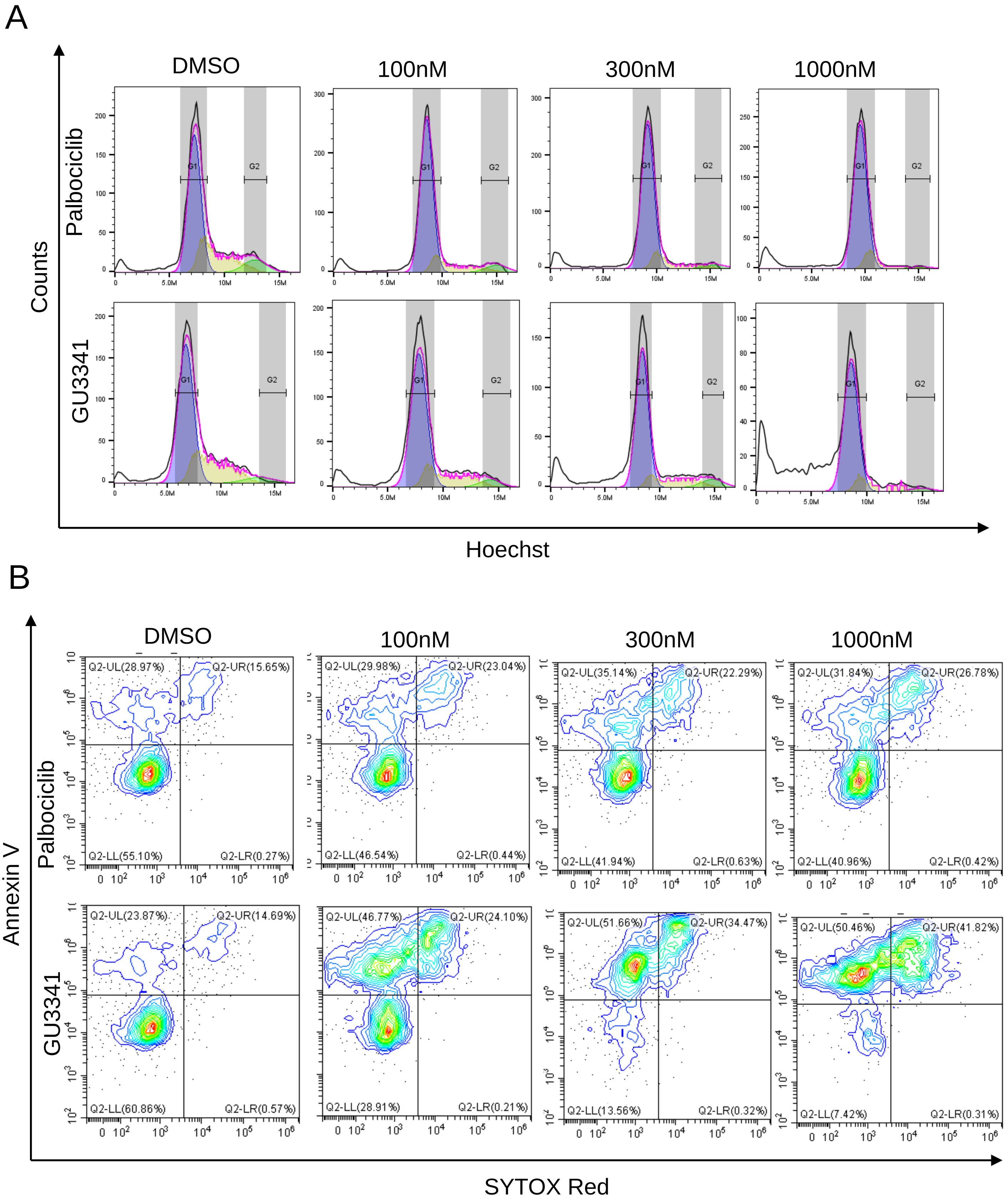
A
DMSO
100nM
300nM
1000nM
Palbociclib
GU3341
Counts
Hoechst
G1
G2
B
DMSO
100nM
300nM
1000nM
Palbociclib
GU3341
Annexin V
SYTOX Red
Q2-UL(28.97%)
Q2-UR(15.65%)
Q2-LL(55.10%)
Q2-LR(0.27%)
Q2-UL(29.98%)
Q2-UR(23.04%)
Q2-LL(46.54%)
Q2-LR(0.44%)
Q2-UL(35.14%)
Q2-UR(22.29%)
Q2-LL(41.94%)
Q2-LR(0.63%)
Q2-UL(31.84%)
Q2-UR(26.78%)
Q2-LL(40.96%)
Q2-LR(0.42%)
Q2-UL(23.87%)
Q2-UR(14.69%)
Q2-LL(60.86%)
Q2-LR(0.57%)
Q2-UL(46.77%)
Q2-UR(24.10%)
Q2-LL(28.91%)
Q2-LR(0.21%)
Q2-UL(51.66%)
Q2-UR(34.47%)
Q2-LL(13.56%)
Q2-LR(0.32%)
Q2-UL(50.46%)
Q2-UR(41.82%)
Q2-LL(7.42%)
Q2-LR(0.31%)

Figure S2

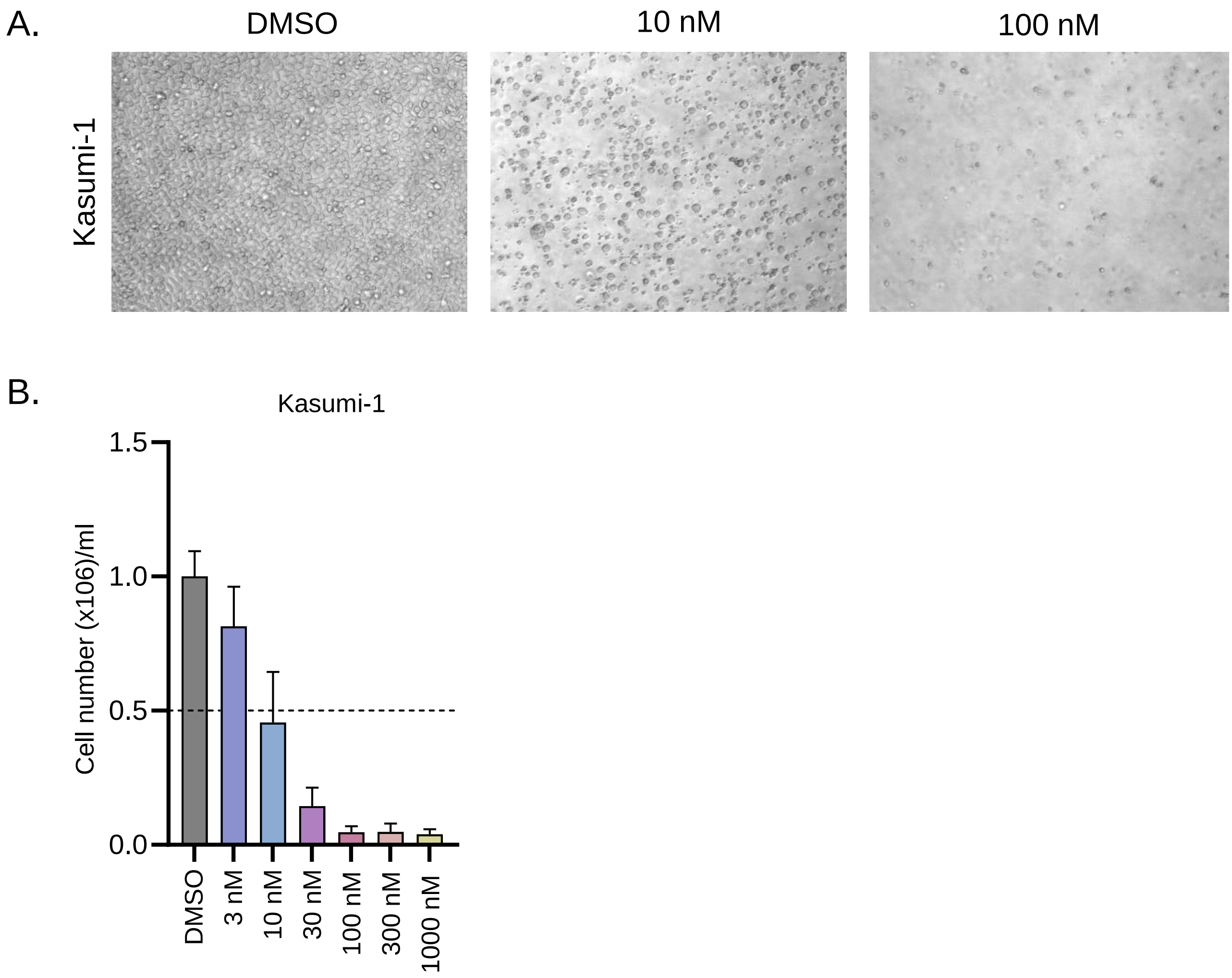
A.
DMSO
10 nM
100 nM
Kasumi-1
B.
Kasumi-1
Cell number (x106)/ml
1.5
1.0
0.5
0.0
DMSO
3 nM
10 nM
30 nM
100 nM
300 nM
1000 nM

Figure S3

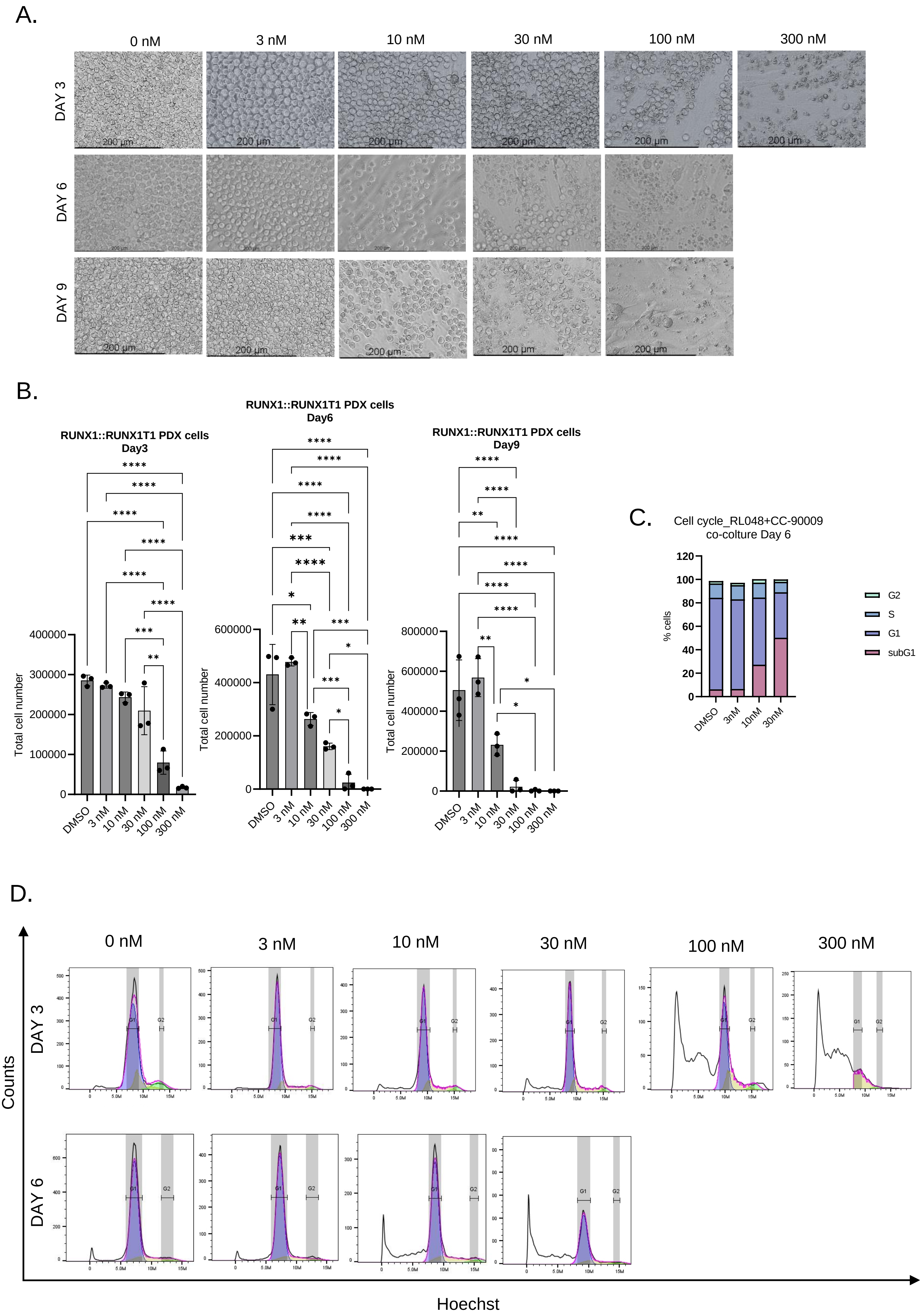
A.
0 nM
3 nM
10 nM
30 nM
100 nM
300 nM
DAY 3
DAY 6
DAY 9
200 µm
B.
RUNX1::RUNX1T1 PDX cells
Day3
RUNX1::RUNX1T1 PDX cells
Day6
RUNX1::RUNX1T1 PDX cells
Day9
Total cell number
DMSO
3 nM
10 nM
30 nM
100 nM
300 nM
C.
Cell cycle_RL048+CC-90009
co-colture Day 6
% cells
G2
S
G1
subG1
DMSO
3nM
10nM
30nM
D.
Counts
Hoechst
DAY 3
DAY 6
0 nM
3 nM
10 nM
30 nM
100 nM
300 nM

Figure S4

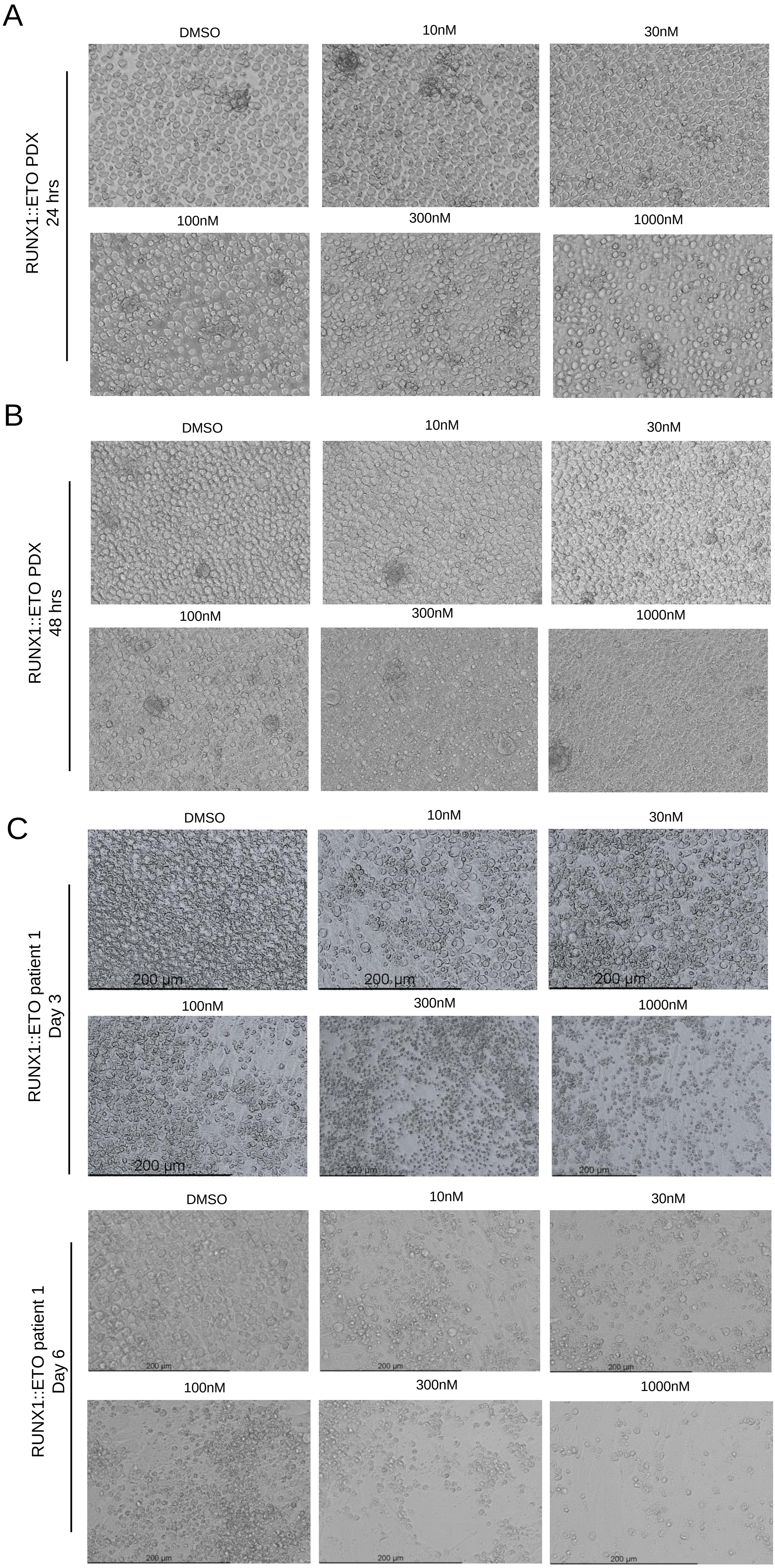
A
DMSO
10nM
30nM
100nM
300nM
1000nM
RUNX1::ETO PDX
24 hrs
B
DMSO
10nM
30nM
100nM
300nM
1000nM
RUNX1::ETO PDX
48 hrs
C
DMSO
10nM
30nM
200 µm
200 µm
200 µm
100nM
300nM
1000nM
200 µm
RUNX1::ETO patient 1
Day 3
DMSO
10nM
30nM
100nM
300nM
1000nM
RUNX1::ETO patient 1
Day 6

Figure S5

A.

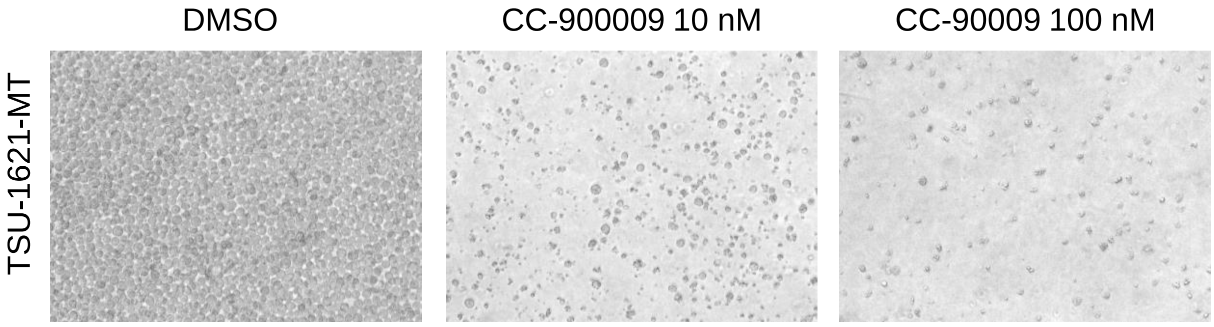

B.

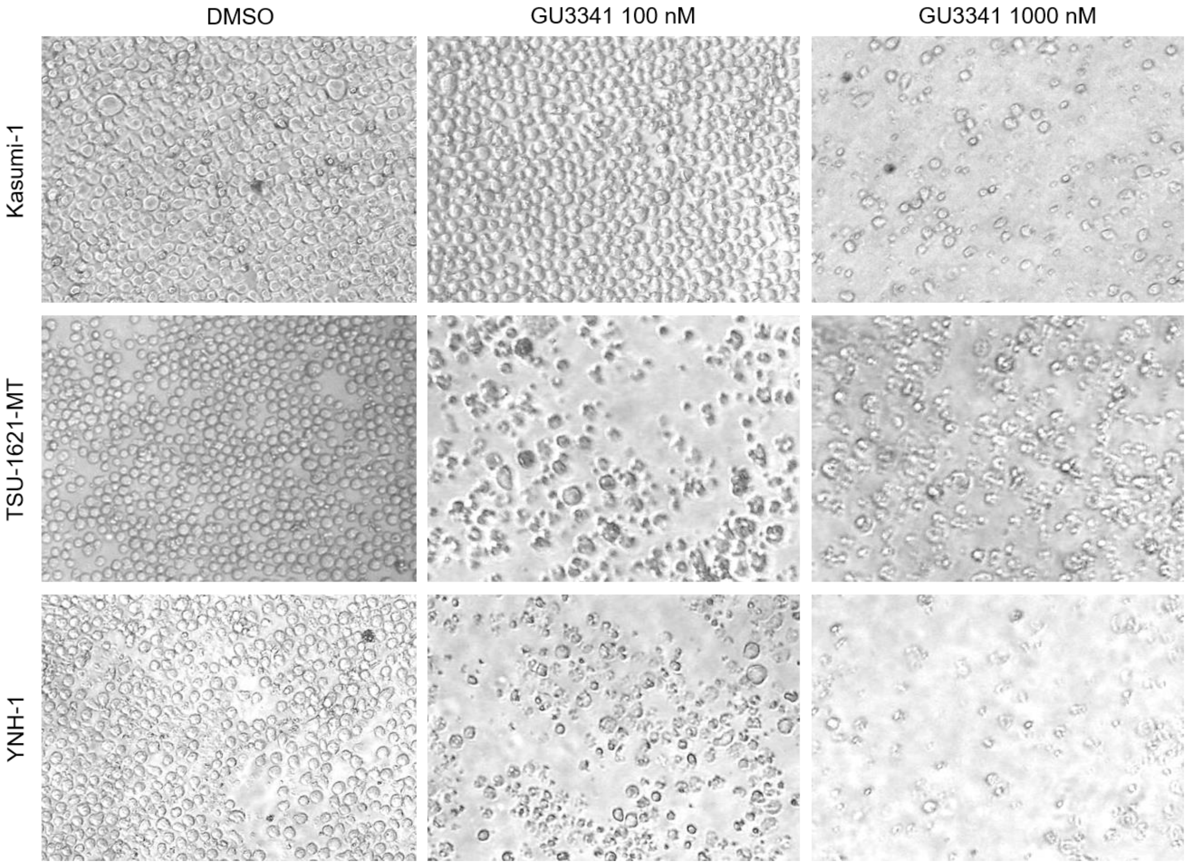

C.

**GU3341**	**ED_{50} (nM) ± SD**
Kasumi-1	164 ± 2
TSU-1621-MT	16 ± 2
YNH-1	29 ± 2

Figure S6

File S1: Full Western Blot membranes (Figure 2A-E)

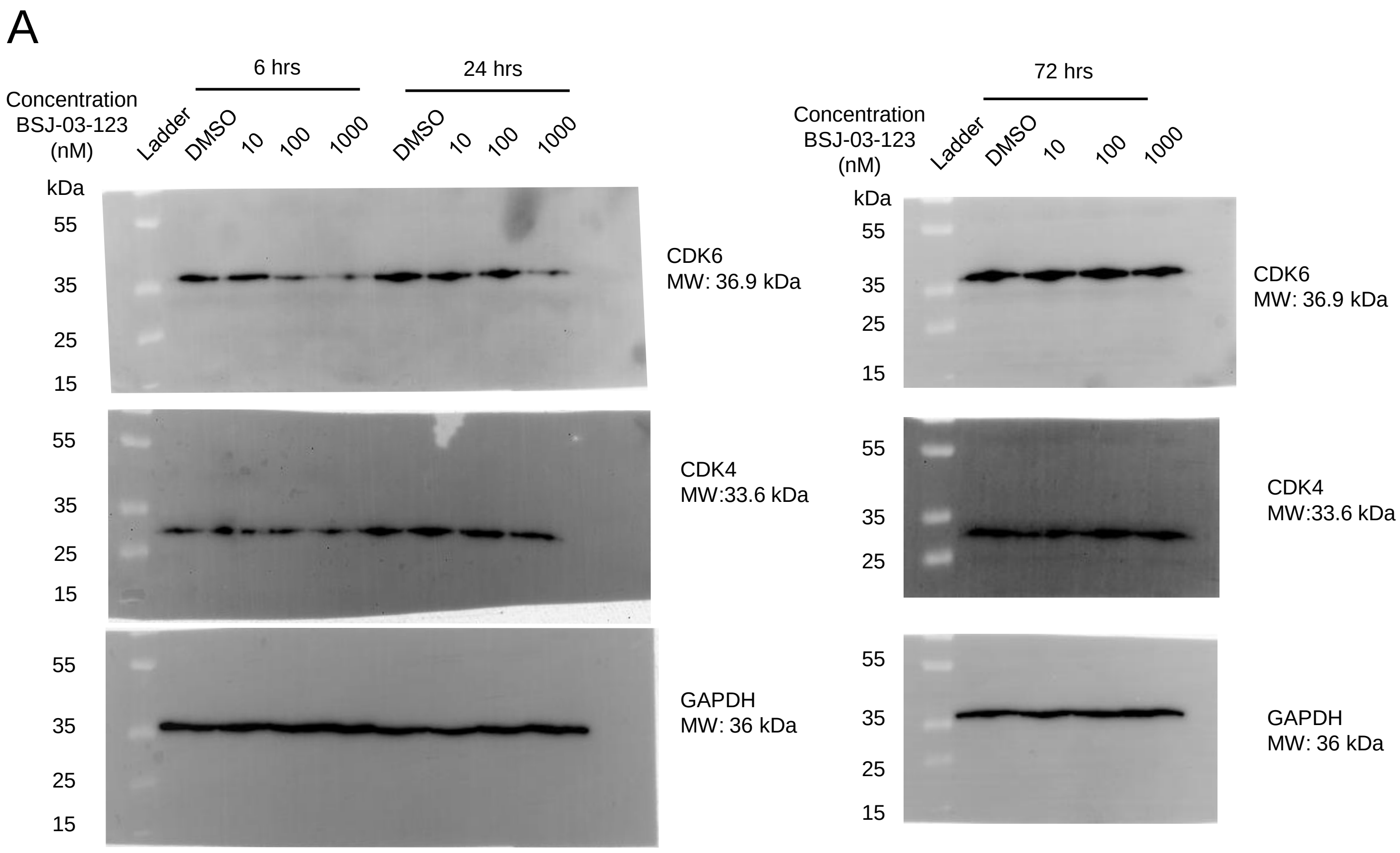

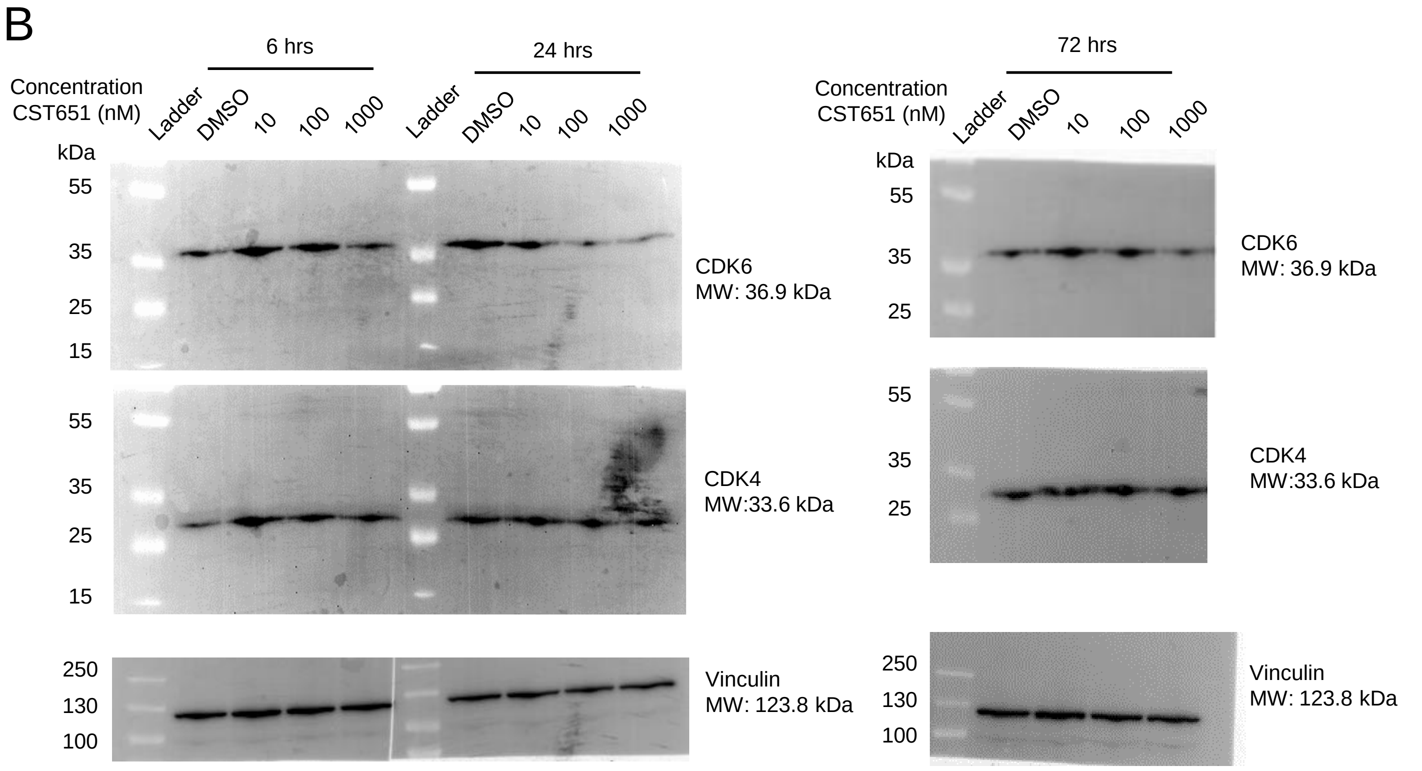

C

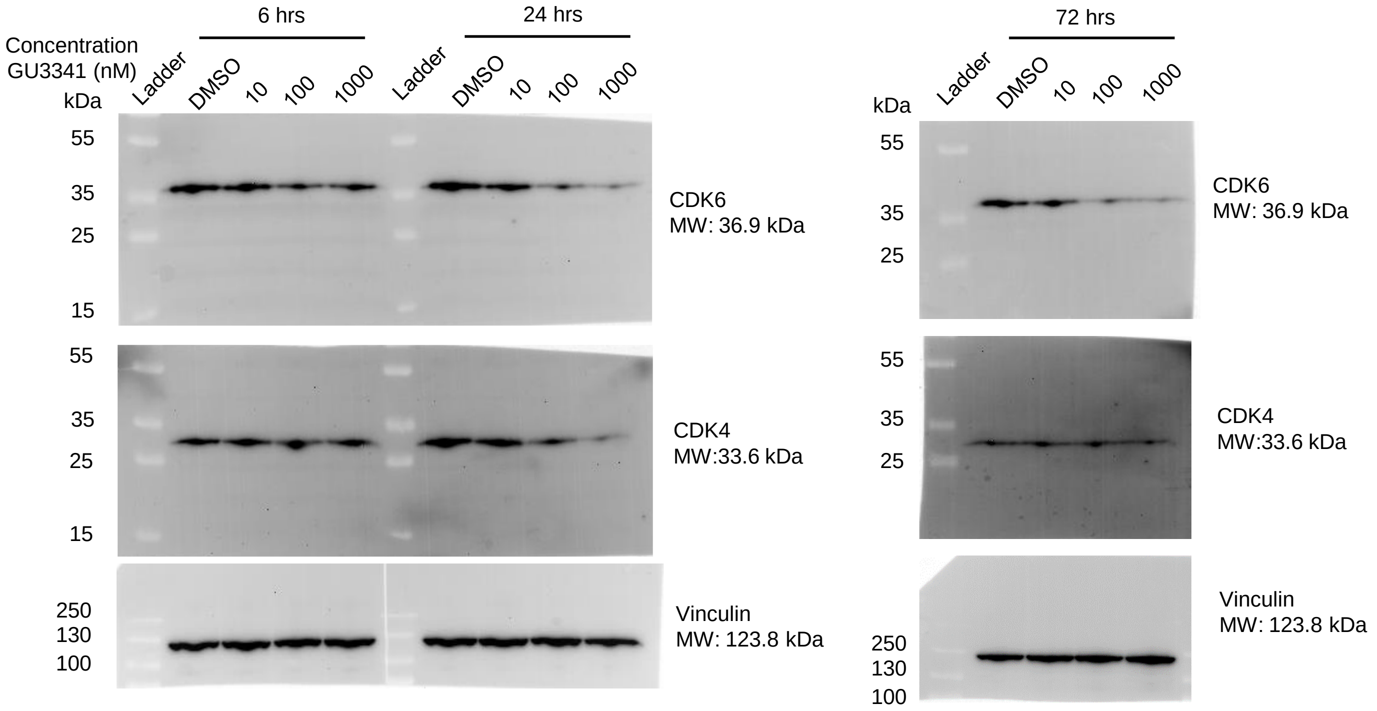

6 hrs
24 hrs
72 hrs
Concentration
GU3341 (nM)
kDa
Ladder
DMSO
10
100
1000
55
35
25
15
CDK6
MW: 36.9 kDa
CDK4
MW:33.6 kDa
250
130
100
Vinculin
MW: 123.8 kDa

D

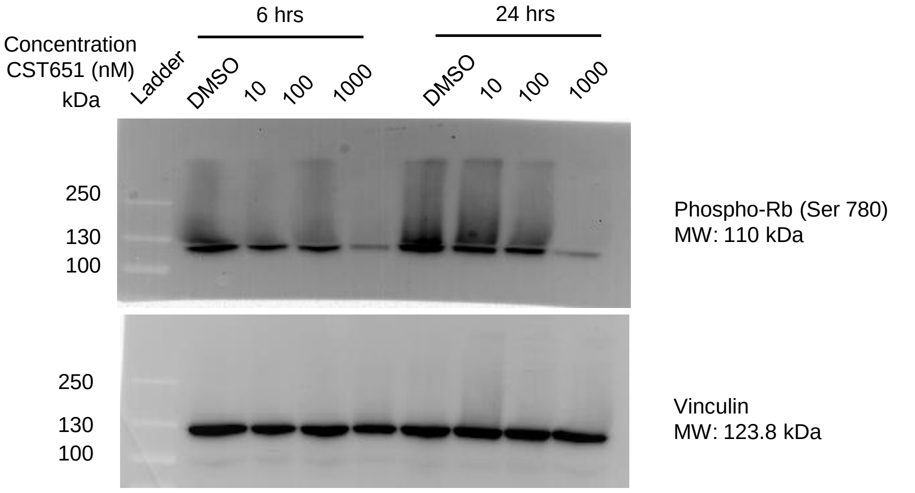

6 hrs
24 hrs
Concentration
CST651 (nM)
kDa
Ladder
DMSO
10
100
1000
250
130
100
Phospho-Rb (Ser 780)
MW: 110 kDa
Vinculin
MW: 123.8 kDa

E

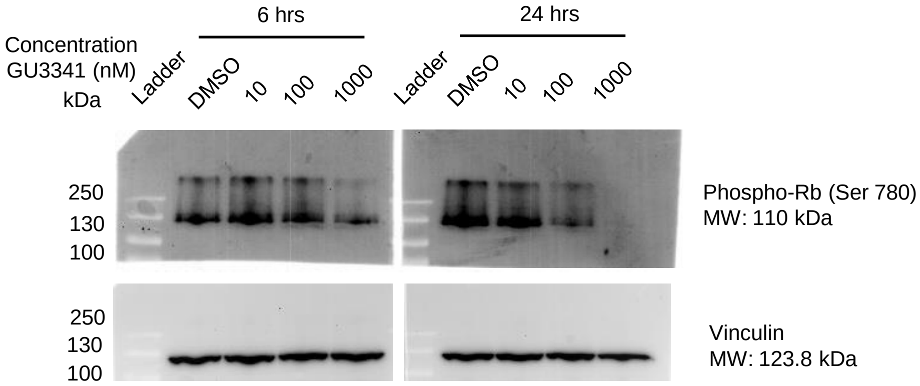

6 hrs
24 hrs
Concentration
GU3341 (nM)
kDa
Ladder
DMSO
10
100
1000
250
130
100
Phospho-Rb (Ser 780)
MW: 110 kDa
Vinculin
MW: 123.8 kDa

File S1: Full Western Blot membranes (Figure 3D)

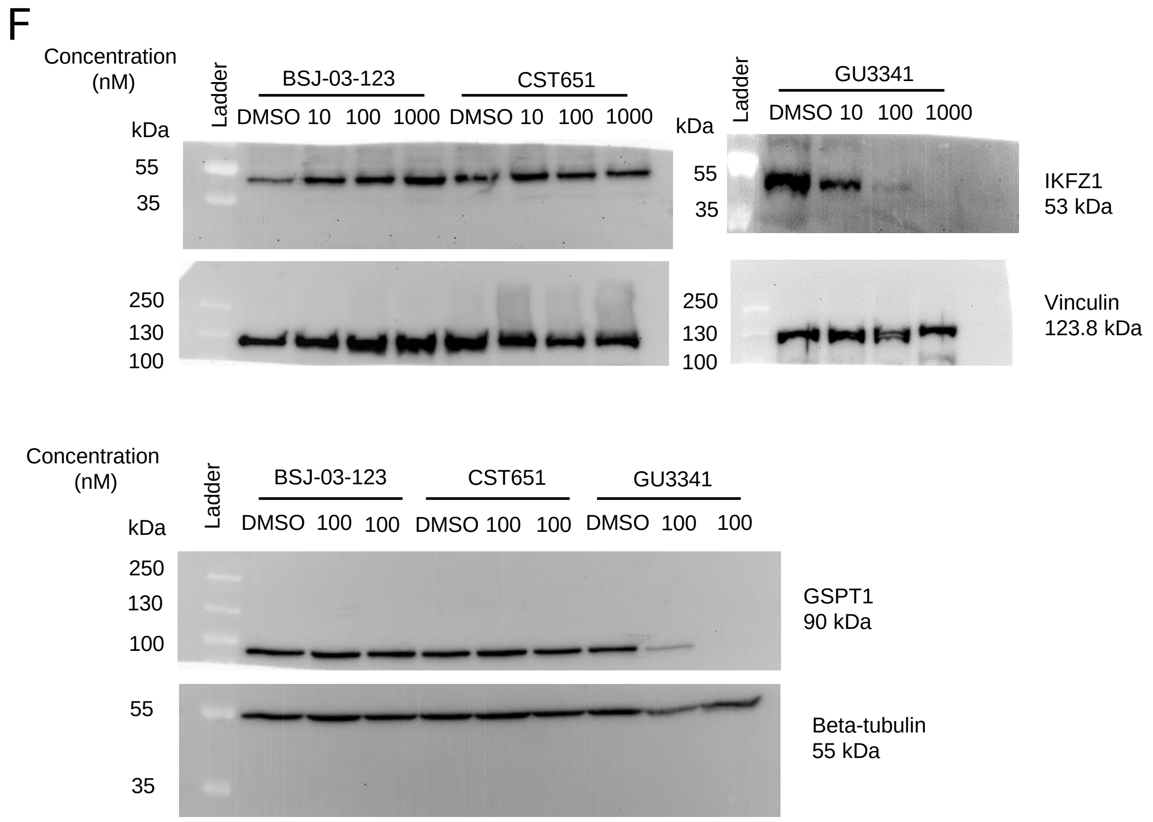

File S1: Full Western Blot membranes (Figure 4C-D, 4H)

G

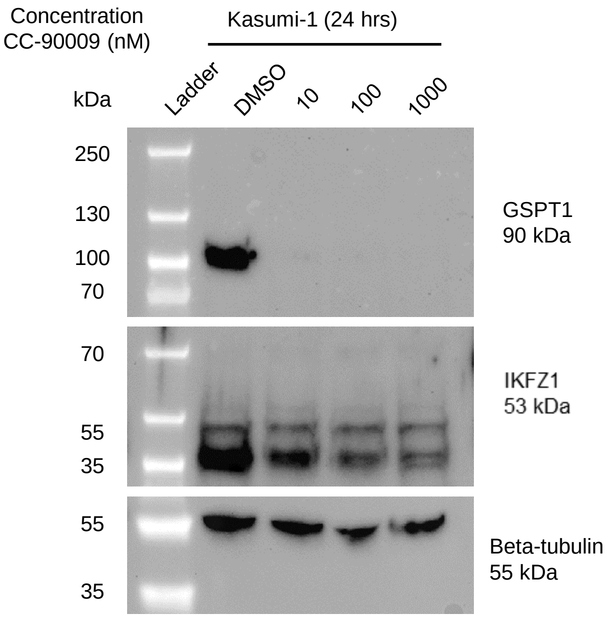

H

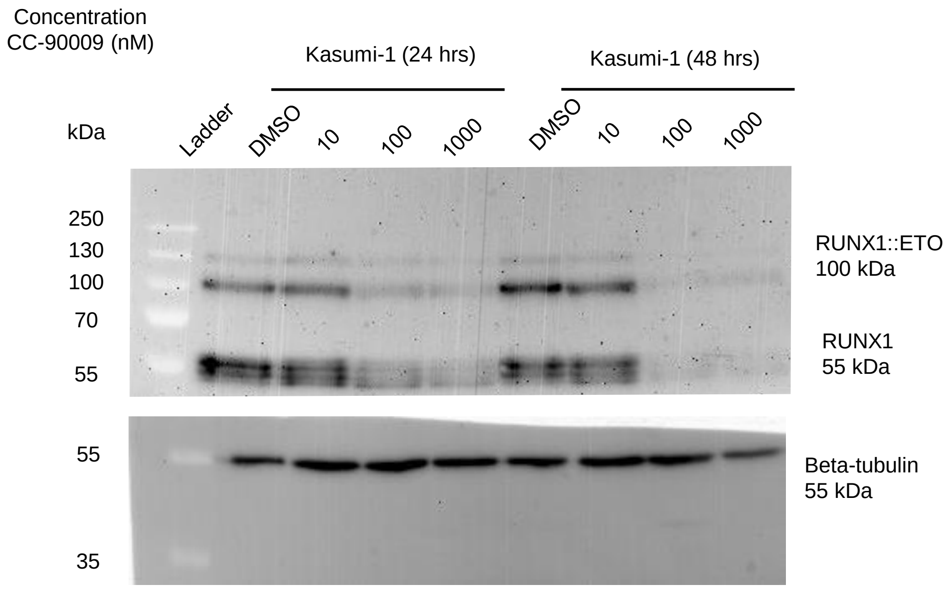

I

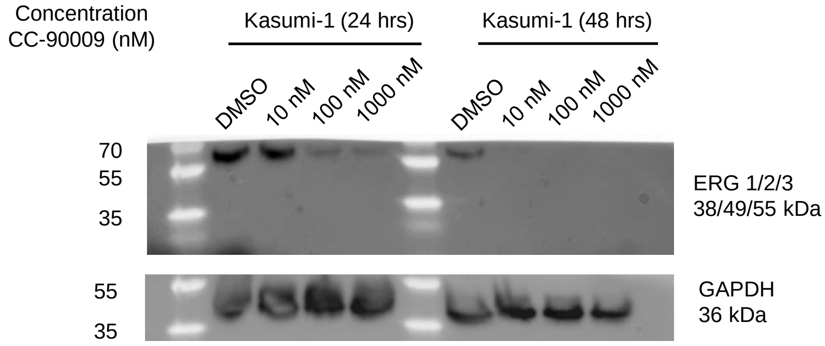

File S1: Full Western Blot membranes (Figure 5E)

L

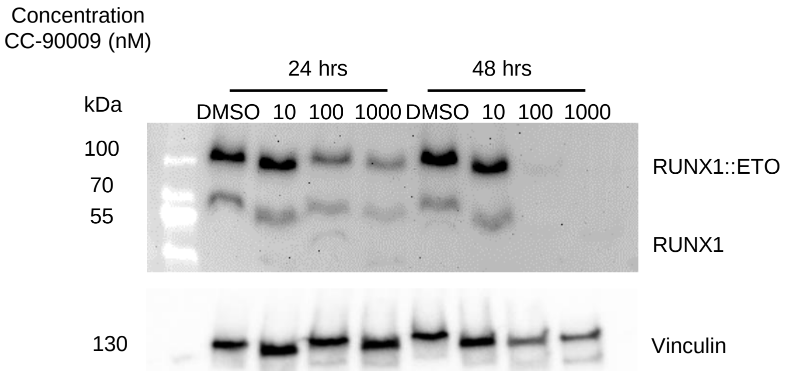

Full Western Blot membranes (Figure 6E)

M

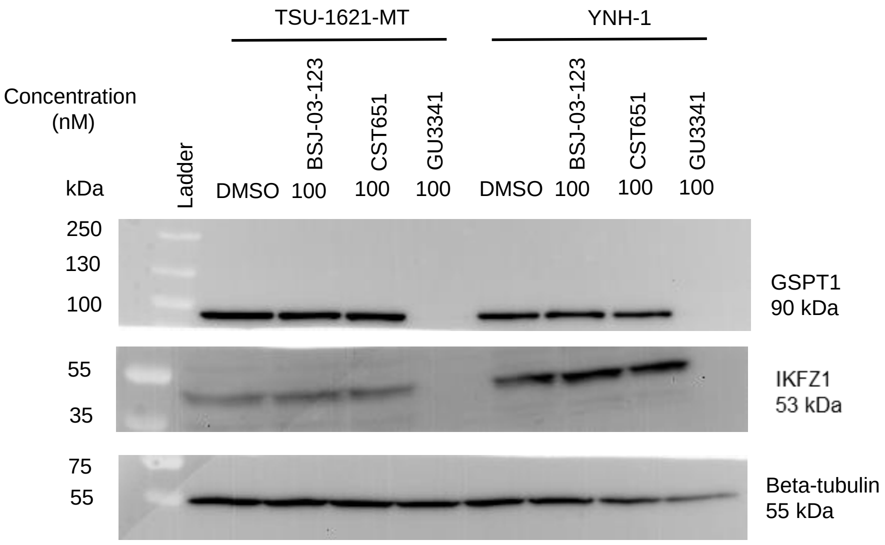

Supplement: Supplementary file 1 [file cancers-17-00211-s001.zip › Supplementary figures_PROTACs.pdf]
